# Supplementary material for: Naturally Occurring Nervonic Acid Ester Improves Myelin Synthesis by Human Oligodendrocytes
Source: Cells. 2019 Jul 29;8(8):786. doi: 10.3390/cells8080786 (PMC6721595; doi:10.3390/cells8080786)
Supplement: Supplementary file 1 [file cells-08-00786-s001.pdf]

Supplementary Table 1. The complete oil composition determined by gas chromatography-mass spectrometry. Fish oil mixture (FOM) and linseed oil (LO), as a negative control, were used to determine the role of the intake of essential lipids on the OPC maturation and the ability of mature OLs to synthesize myelin proteins and sphingomyelin. The red color indicates acid esters that can directly bind to sphingosine in the human brain [34]. Blue color indicates fatty acids that possess immunomodulatory properties [46-48].

	Lipid acid	*Linseed oil [%]*	*Fish oil mixture [%]*
**1**	Myristic Acid Me ester (C14:0)	n.d.	1.05
**2**	Pentadecanoic, methyl ester	n.d.	0.25
**3**	Palmitic Acid Me ester (C16:0)	2.25	9.46
**4**	Palmitoleic Acid Me ester (C16:1)	n.d.	2.97
**5**	Tridecanoic acid, methyl ester	n.d.	0.50
**6**	Stearic Acid Me ester (C18:0)	3.69	3.02
**7**	Oleic Acid Me ester (C18:1n9c)	16.46	16.98
**8**	Elaidic Acid Me ester (C18:1n9t)	n.d.	4.93
**9**	Linoleic Acid Me ester (C18:2n6c)	13.43	n.d.
**10**	Linolelaidic Acid Me ester (C18:2n6t)	n.d.	0.68
**11**	alfa-Linolenic Acid Me ester (C18:3n3)	64.17	n.d.
**12**	cis-11,14,17-Eicosatrienoic Acid Me ester (C20:3n3)	n.d.	0.30
**13**	Stearidonic acid Me ester (C18:4n3)	n.d.	1.37
**14**	Arachidic Acid Me ester (C20:0)	n.d.	0.26
**15**	cis-11-Eicosenoic Acid Me ester (C20:1n9)	n.d.	9.05
**16**	Arachidonic Acid Me ester (C20:4n6)	n.d.	0.71
**17**	Stearidonic acid Me ester (C18:4n3)	n.d.	0.82
**18**	cis-5,8,11,14,17-Eicosapentaenoic Acid Me ester (C20:5n3)	n.d.	16.92
**19**	cis-13-docosenoic acid Me ester (C22:1n9)	n.d.	5.39
**20**	cis-4,7,10,13,16,19-Docosahexaenoic Acid Me ester (C22:6n3)	n.d.	9.31
**21**	Nervonic Acid Me ester (C24:1n9)	n.d.	12.76
**22**	Squalene	n.d.	3.28

n.d. – non detectable (<0.3%)
